# COVID-19, SARS-CoV-2 Vaccination, and Human Herpesviruses Infections

**DOI:** 10.3390/vaccines11020232

**Published:** 2023-01-20

**Authors:** Peter A. C. Maple

**Affiliations:** 1 Mental Health and Clinical Neuroscience Academic Unit, University of Nottingham, Nottingham NG7 2UH, UK; eastyorksmicrobiol@gmail.com or peter.maple@nottingham.ac.uk; 2 Department of Neurology, Nottingham University Hospitals NHS Trust, Nottingham NG7 2UH, UK

**Keywords:** COVID-19, SARS-CoV-2 vaccination, cytomegalovirus, herpes simplex virus reactivation, Epstein–Barr virus reactivation, herpes zoster

## Abstract

There are several human herpesviruses. A common characteristic of infection by these viruses is latency, by which the virus assumes a non-replicative state, subverting the attentions of the host’s immune response. In immunocompetent hosts, herpesviruses are immunologically controlled, although periodic virus shedding can occur. In situations where immunological control is lost, herpesviruses can reactivate and produce clinically apparent disease. It is now becoming apparent that COVID-19 or exposure to COVID-19 vaccines can exert several effects on the immune system. The pandemic of COVID-19 shows no sign of abating, with new severe acute respiratory syndrome coronavirus-2 (SARS-CoV-2) variants continuing to evolve. Several COVID-19 vaccines have been developed, and much of the world’s population has either experienced COVID-19 or been vaccinated against it. There are an increasing number of reports of associations between herpesvirus infections or reactivations and COVID-19 or COVID-19 vaccination. For instance, a positive cytomegalovirus serostatus may indicate a greater likelihood of severe COVID-19, and herpes simplex virus reactivation may be linked to increased mortality. Epstein–Barr virus reactivation appears to be associated with post-acute sequelae of COVID-19. Finally, herpes zoster has been reported to be associated with COVID-19 vaccination. This brief narrative review will provide several insights into associations between herpesvirus infections or reactivations and COVID-19 or SARS-CoV-2 vaccination.

## 1. Introduction

COVID-19 (coronavirus disease 2019) is the current World Health Organization-approved term used to describe the clinical syndrome [1,2] associated with infection by severe acute respiratory syndrome coronavirus-2 (SARS-CoV-2). Initially, the most common signs and symptoms included fever, dry cough, and dyspnoea [1,2]. Clinical presentations have ranged from asymptomatic to life-threatening severe acute respiratory syndrome [3,4]. A global pandemic of COVID-19 was declared by the World Health Organization in March 2020 [5] and continues to the present day. Over this period of time, mutations of SARS-CoV-2 have resulted in waves of infection of several variants of the virus [6]. These variants have displayed differing capacities for spread and produced severe disease in both vaccinated and non-vaccinated populations [7,8], particularly as a consequence of new mutations in the SARS-CoV-2 spike receptor-binding domain, potentially enabling evasion of neutralizing antibody responses. SARS-CoV-2 vaccination is a fundamental strategy for reducing COVID-19 and, to date, several vaccines have been licensed for use, while others are in the late stages of development [9]. It is becoming increasingly realised that vaccination will be a long-term measure for controlling the COVID-19 pandemic and, similarly to influenza, regular boosting will be required [10].

Nine human herpesviruses have been described. According to recently updated nomenclature [11], these are *Human alphaherpesvirus 1* (herpes simplex virus type 1), *Human alphaherpesvirus 2* (herpes simplex virus type 2), *Human alphaherpesvirus 3* (varicella-zoster virus), *Human gammaherpesvirus 4* (Epstein–Barr virus), *Human betaherpesvirus 5* (human cytomegalovirus), *Human betaherpesvirus 6A* (human herpesvirus 6A), *Human betaherpesvirus 6B* (human herpesvirus 6B), *Human betaherpesvirus 7* (human herpesvirus 7), and *Human gammaherpesvirus 8* (Kaposi’s sarcoma herpesvirus). Throughout this review, historical nomenclature/common names will be used for the human herpesviruses. A uniform characteristic of human herpesviruses is their capacity to establish long-term or life-long immunopathological relationships with their human hosts [12]. Following primary infection, human herpesviruses are not eradicated by the host’s immune response, and virus infection is maintained in various cell types in a mostly non-replicative state (latent infection). Should the host’s immune control of virus infection be diminished, for example, by immune senescence or iatrogenic events (e.g., induced immunosuppression for transplantation) or infection by other viruses (e.g., HIV), human herpesviruses can reactivate, potentially causing severe disease (Table 1).

SARS-CoV-2 infection or vaccination evokes an immune response; the interaction of the virus with the human host is complex and remains to be fully determined [22,23]. Several different pathologies have been identified following SARS-COV-2 infection, e.g., asymptomatic infection [24], acute respiratory distress syndrome with cytokine storm [3,4], and post-acute sequelae of COVID-19 [25], commonly described as “long COVID”. In this review, several associations of human herpesvirus infections following SARS-CoV-2 infection or vaccination will be described.

## 2. COVID-19 and Human Herpesviruses Reactivations

There have been several reports of systemic or pulmonary reactivation of Herpes Simplex Virus (HSV-1) in critically ill COVID-19 patients (Table 2). This topic has recently been reviewed by Giacobbe et al. [26], who reviewed seven studies of HSV-1 reactivation in critically ill COVID-19 patients together with relevant immunology and clinical implications. These authors noted that the prevalence of HSV-1 reactivation may be as high as >50%, but with a large heterogeneity across studies that is potentially attributable to a lack of standardization. Specifically, some reports have noted the clinical significance of HSV-1 reactivations to be equivocal; for example, Luyt et al. [27] have reported a 50% rate of HSV lung reactivation in 145 patients with severe COVID-19 pneumonia requiring invasive mechanical ventilation but did not observe any impact on patient outcomes. In an attempt to clarify the association between HSV-1 reactivation and mortality, Meyer et al. [28] conducted an observational study of 153 critically ill COVID-19 patients using prospectively collected data and samples. In this study [28], 26.1% patients had confirmed HSV-1 reactivation, and day-60 mortality was higher in patients with HSV-1 reactivation (57.5%) versus without (33.6%).

There is evidence [29] that herpes zoster due to varicella-zoster virus (VZV) reactivation has increased during the COVID-19 pandemic, which may possibly be related to the lymphopenia commonly associated with SARS-CoV-2 infection [30,31]. Salim Ali Algaadi [30] recently reviewed several case reports of herpes zoster associated with COVID-19, with the conclusion that there is a potential causal relationship between COVID-19 and subsequent herpes zoster. Unfortunately, most of the evidence for this phenomenon is derived from case reports, and there is a need for further epidemiological studies.

Results from a large Italian observational study of COVID-19 patients with moderate to severe acute respiratory distress syndrome [32] have shown cytomegalovirus (CMV) viraemia/reactivation in 20.4% of patients studied (Table 2). There have been several reports describing CMV reactivation with gastrointestinal tract involvement [33]. It has been suggested by Alanio et al. [34] that latent CMV infection is associated with an increased risk of COVID-19-related hospitalisation. These authors [34] demonstrated that CMV seropositivity was associated with more than twice the risk of hospitalisation due to SARS-CoV-2 infection. Furthermore, a subset of patients was immune profiled, revealing altered T cell activation profiles potentially indicative of CMV-mediated immune phenomena influencing the outcome and severity of SARS-CoV-2 infection. Other studies—for example, Weber et al. [35]—have also identified CMV seropositivity as a potential novel risk factor for severe COVID-19 (Table 2). Finally, Pius-Sadowska et al. [36] reported higher plasma concentrations of chemokines CXCL8 and CCL2, together with CMV-seropositivity, to be potential prognostic factors for severe COVID-19 disease.

Epstein–Barr virus (EBV) reactivation has frequently been detected in COVID-19 patients [37,38], and in some reports [39,40], it has been associated with greater morbidity and mortality. For instance, Chen et al. [39] reported a high incidence of EBV reactivation in COVID-19 patients, which was associated with fever and increased inflammation. In another study, Xie et al. [40] reported 17 (13.3%) of 128 COVID-19 patients to show evidence of EBV reactivation. This group also had higher day-14 and day-28 mortality rates compared to the EBV non-reactivated group. Cases of human herpesvirus-6 reactivation or coinfection have also been reported in association with COVID-19 [41,42]. In both studies [41,42], HHV-6 reactivation was detected, but there was no evidence of an association with COVID-19 disease severity or mortality.

**Table 2:** Selected studies of herpesviruses reactivations in severely ill COVID-19 patients.

Herpesvirus Reactivation and Study Reference	Total Patients and Clinical Characteristics of Study Group	Results	Conclusions/Comments
HSV-1 Luyt et al. [27]	Retrospective monocentric cohort study of 145 patients with severe COVID-19 pneumonia requiring invasive mechanical ventilation.	Among 145 COVID-19 patients, a total of 50% and 42% had HSV and CMV lung reactivations, respectively, compared to 63% and 28% HSV and CMV lung reactivations in a control group of 89 influenza patients.	HSV and CMV lung reactivations are frequent in COVID-19 patients subject to invasive mechanical ventilation; however, they are no more frequent than in controls with influenza. HSV and CMV reactivations were defined by a positive PCR test result in bronchoalveolar lavage fluid samples or whole blood samples.
HSV Meyer et al. [28]	Observational study using prospectively collected data, as well as HSV-1 blood and respiratory samples from 153 critically ill COVID-19 patients admitted to a regional intensive care unit (ICU) for at least 48 h, from February 2020 to February 2021.	Respiratory and blood samples were tested from 61/153 (39.9%) and 146/153 (95.4%) patients, respectively. On the basis of respiratory sample testing, HSV PCR was positive in 19/61 (31.1%) of patients, and on the basis of blood sample testing, HSV PCR was positive in 36/146 (24.7%) of patients.	Overall, 40/153 (26.1%) patients had an HSV PCR positive sample. HSV reactivation was defined as testing positive by HSV PCR. Day-60 mortality in the whole cohort was 39.9% higher in patients with HSV-1 reactivation (57.5% versus 33.6% in patients without HSV-1 reactivation, *p* = 0.001).
CMV Gatto et al. [32]	Observational study using prospectively collected data of all the patients with moderate to severe acute respiratory distress syndrome admitted to three COVID-19 ICUs at the University Hospital of Modena over the period from 22 February 2020 to 21 July 2021.	A total of 431 patients met the study’s inclusion criteria. COVID-19 was confirmed by laboratory detection of SARS-CoV-2. CMV reactivation was evidenced in whole blood samples by CMV PCR with a cut-off of >62 IU/mL.	Blood CMV reactivation was detected in 88/431 (20.4%) patients, with a median onset of 17 days following ICU admission. Patients with CMV reactivation had prolonged hospital stays and a higher mortality rate than patients without reactivation. CMV reactivation was not independently associated with higher mortality.
CMV and HSV Weber et al. [35]	National German COVID-19 bio-sample and data banks were used to retrospectively analyse the CMV and HSV status of patients. Serum samples were collected from patients who experienced mild (n = 101), moderate (n = 130), or severe to critical (n = 80) COVID-19.	CMV seropositivity was 43.6% in cases of mild COVID-19, 72.3% in cases of moderate COVID-19, and 77.5% in cases of severe to critical COVID-19. HSV seropositivity was 71.3%, 93.8%, and 96.2%, respectively, in the same groups.	Patients aged <60 years with severe COVID-19 had a very high prevalence of CMV seropositivity. CMV seropositivity, unlike HSV, might be a strong biomarker for identifying patients <60 years with a higher risk of developing severe COVID-19, particularly in the absence of other co-morbidities.
EBV Chen et al. [39]	A retrospective, single-centre study from 9 January 2020 to 29 February 2020: a total of 188 hospitalised patients were recruited with PCR-confirmed SARS-CoV-2 infection.	EBV serology was available for 78 patients, and 11 failed to meet the study inclusion criteria. Of the remaining 67 patients, 37 (55.2%) had laboratory evidence of EBV reactivation. EBV viral load testing was not undertaken.	Patients with laboratory evidence of EBV reactivation had a 3.09-fold risk of having a fever symptom. C-reactive protein levels were significantly elevated in patients with EBV reactivation.
EBV Xie et al. [40]	Retrospective, single-centre, observational study of ICU admissions over the period from 31 January 2020 to 27 March 2020.	145 critically ill patients with SARS-CoV-2/PCR-confirmed COVID-19 were recruited into the study, and 128 met the study’s inclusion criteria. EBV viral load testing (≥500 copies/mL) and serology were used as evidence of EBV reactivation.	Patients with EBV reactivation had higher (29.4%) day-14 and day-28 mortality rates compared to 7.8% and 10.9%, respectively, for patients without EBV reactivation. Patients with evidence of EBV reactivation showed more severe symptoms and received more immunosupportive treatment.
HHV-6 Lino et al. [42]	Retrospective, single-centre study of hospitalised patients with moderate to severe COVID-19	173 patients with suspected COVID-19 were recruited, of which 60 had a positive PCR test for SARS-CoV-2. Of these 60 confirmed cases, 13/60 (21.7%) were also had positive PCR tests for HHV-6.	HHV-6 reactivation did not impact general mortality.

Abbreviations: CMV = cytomegalovirus, EBV = Epstein–Barr virus, HHV-6 = human herpesvirus-6, HSV = herpes simplex virus, ICU = intensive care unit, PCR = polymerase chain reaction, VZV = varicella-zoster virus.

## 3. Human Herpesviruses Infections and Long COVID

Post-acute COVID-19 syndrome (PASC), commonly called “Long COVID”, is loosely defined as a diverse collection of clinical presentations continuing or appearing four weeks beyond initial SARS-CoV-2 infection [43]. These presentations may include pulmonary (e.g., breathlessness, decreased exercise capacity), cardiovascular (e.g., palpitations, chest pain), neurological (e.g., fatigue, myalgia), renal, endocrine, gastrointestinal, and dermatologic elements, either alone or in combination. The defining symptoms of COVID-19 are headaches and loss of the senses of taste and smell, which have led to suggestions that SARS-CoV-2 is neuroinvasive [44]. Persistent cognitive symptoms, including a syndrome of persistent cognitive impairment (“brain fog”) characterised by impaired attention, concentration, memory, speed of information processing, and executive function have been widely reported following SARS-CoV-2 infection [45]. Few studies have investigated the potential association of human herpesviruses reactivation with the neurological manifestations of PASC; however, recently published studies have suggested that EBV reactivation may be linked with PASC. Gold et al. [46] have reported EBV reactivation in 66.7% of long COVID subjects, with the conclusion that many long COVID symptoms may be the result of COVID-19 inflammation-induced EBV reactivation. These findings [46] were based on an analysis of 185 subjects who responded to online advertisements and met the initial study inclusion criteria, including a documented history of COVID-19. Further sifting of study participants was then undertaken to establish four study groups: two primary (long-term) study groups and two secondary (short-term) study groups. These study groups comprised a long-term/long COVID group (30 subjects who had tested positive for COVID-19 at least 90 days prior to enrolment and who had reported at least one long COVID symptom) and a long-term/control group (20 subjects who were COVID-19 positive, as for the long-term/long COVID group, but who reported no long COVID symptoms. Blood samples from these individuals were tested for serological markers of EBV reactivation, which were found to be present in 20/30 (66.7%) long-term/long COVID group subjects and 2/20 (10%) long-term/control subjects. In a separate study [47], Rohrhofer et al. analysed whether long COVID fatigue is triggered by SARS-CoV-2 persistence in the gastrointestinal or respiratory tract following SARS-CoV-2 infection. In this study [47], stool and throat washings were tested for SARS-CoV-2 RNA, and stool and throat washings in addition to blood samples were tested for EBV DNA. Two patient groups were tested, a long COVID group of 30 patients (characterised by persistent fatigue, post-exertional malaise, autonomic dysfunction, and/or orthostatic intolerance) and a control group of 20 age and sex-matched patients (fully recovered from SARS-CoV-2 infection). EBV DNA was detected in throat washings from 15/30 (50%) of long COVID patients and 4/20 (20%) controls. It is highly evident that further epidemiological and mechanistic studies are required to determine the potential associations between long COVID and EBV reactivation.

## 4. SARS-CoV-2 Vaccination and Human Herpesviruses Infections

Herpes zoster following COVID-19 vaccination has been widely reported [48,49,50,51,52,53], although conclusive evidence of a direct link is lacking. Table 3 summarises these selected reports. There is always the possibility, in those cases where zoster manifested a short time following COVID-19 vaccination, that it was an unrelated event that occurred at the same time. Data from retrospective analyses of adverse event databases following COVID-19 vaccination [54,55,56] or case-control studies [57] support an increased risk of developing zoster following COVID-19 vaccination. In a recent systematic review [58] of evidence of VZV reactivation or infection following SARS-CoV-2 vaccination, Martinez-Reveijo et al. [58] concluded that the occurrence of VZV reactivation is clinically relevant. In their analysis [58], 55 reports met the inclusion criteria. VZV manifestations were documented in 179 (82.1%) subjects following SARS-CoV-2 vaccination and in 39 (17.9%) patients with COVID-19. In the vaccinated group, the median age was 56.5 years, and 56.8% of vaccinees were female. Most (104/125 = 83.2%) vaccinated subjects were immunocompetent and the majority (151/179 = 84.4%) had received mRNA vaccine. Nearly all (c. 90%) of the reactivations were non-serious, with most manifestations being skin rashes showing dermatomal distributions which appeared within 10 days of the first vaccine dose. Other studies [59] have failed to identify an association between COVID-19 vaccination and herpes zoster. Akpandak et al. [59] undertook a cohort study using a self-controlled risk interval (SCRI) design to compare the risk of herpes zoster in a risk interval of 30 days after COVID-19 vaccination, or up to the date of the second vaccine dose. Among 2,039,854 subjects who received any dose of a COVID-19 vaccine during the study period (11 December 2020 to 30 June 2021), a subset of 1451 patients was identified who had herpes zoster in either the risk or control period. Following SCRI analysis, COVID-19 vaccination was not associated with an increased risk of herpes zoster.

## 5. Conclusions

Human herpesvirus infections are widespread in populations worldwide, and by virtue of their capacity to establish latency [12], they have established a unique relationship with their human hosts in which infection is maintained for life. Latency is the outcome of virus host adaptation, in which herpesvirus genomes are maintained in a non-replicative state, hidden from the host’s immune response. Changes in the host’s immune status can result in a failure of the host to suppress herpesvirus replication with consequent disease manifestations [45,60,61]. Infection by severe acute respiratory syndrome coronavirus-2 (SARS-CoV-2) can have several clinical outcomes depending upon the host’s immunological response to infection [62], and treatments for COVID-19 can also be immunosuppressive. In this short review, evidence has been provided that SARS-CoV-2 infection can lead to herpesvirus reactivations—a phenomenon that has been observed with other respiratory virus infections [27]. Unfortunately, there is a general lack of data addressing this topic and a lack of prospectively controlled epidemiological studies with which to establish a firm evidence base. Furthermore, detailed mechanistic studies of how SARS-CoV-2 infection may contribute to herpesvirus reactivation or how herpesvirus reactivation (or previous infection) may impact SARS-CoV-2 infection are lacking. Clinicians need to be alerted to these potential complications; particularly as effective treatments are available for many herpesvirus infections. Finally, herpesviruses have been shown to be associated with several autoimmune conditions, particularly multiple sclerosis [63]. There is emerging evidence that SARS COV-2 infection can generate autoimmune processes [60,64] and Epstein–Barr virus reactivation triggered by COVID-19-related hyperinflammatory responses may adversely impact multiple sclerosis disease progression [65].

Understanding how SARS-CoV-2 impacts the human immune system is an ongoing challenge, particularly as the virus continues to evolve. Most recently, there has been the emergence and spread of new omicron subvariants which have potentially adapted to evade the humoral immune response, including that generated by vaccination. The new subvariants of current concern are BQ.1, BQ.1.1, XBB, and XBB.1, and the rapid rise of these subvariants and their extensive array of spike mutations (which have been shown in vitro to confer significantly reduced susceptibility to neutralizing antibodies) present serious threats to the efficacy of current COVID-19 vaccines [66]. Other reports of particular interest in relation to the interactions SARS-CoV-2 has with its human hosts include those by Stein et al. [67] and Wang et al. [68]. In what is probably the most comprehensive study to date, Stein et al. [67] undertook autopsies on 44 patients who died with COVID-19 in order to investigate the cellular tropism, replication competence, persistence, and evolution of SARS-CoV-2 in humans. They found that SARS-CoV-2 was widely distributed, predominantly among patients who died with severe COVID-19, and that virus replication was present in multiple respiratory and non-respiratory tissues, including the brain, early in infection. Furthermore, they presented definitive evidence that SARS-CoV-2 was capable of infecting and replicating within the human brain. It is well known that SARS-CoV-2 infection is associated with neurological sequelae [23,43], both in the acute stages (e.g., loss of senses of smell or taste, headache) and post-acute stages (e.g., cognitive impairment), and Wang et al. [68] have presented evidence of an association of COVID-19 with new-onset Alzheimer’s disease. In their retrospective cohort study [68] of 6,245, 282 people aged 65 years or more, they identified that people with COVID-19 were at significantly increased risk for diagnosis of Alzheimer’s disease within 360 days after their initial COVID-19 diagnosis. A potential explanation for this phenomenon is that COVID-19 results in repeated reactivation of HSV-1 in the brain, with the subsequent accumulation of damage and the eventual development of Alzheimer’s disease [69].

In this brief narrative review, several insights into associations between herpesvirus infections or reactivations and COVID-19 or SARS-CoV-2 vaccination have been described. This is very much a developing area of knowledge in need of basic scientific mechanistic studies, together with appropriate clinical and epidemiological studies. Unfortunately, due to the rapid and ongoing evolution of SARS-CoV-2, we may be rapidly approaching an era where current vaccine-induced immunity is mostly ineffective and fundamentally new vaccines are needed [70]. An awareness of herpesvirus interactions with COVD-19 is important, as it may influence treatment decisions and patient outcomes; for example, the knowledge of CMV seropositivity as an indicator of an increased likelihood of more severe disease may influence clinical decision-making. Similarly, a greater understanding of the role of EBV reactivation in post-acute sequelae of COVID-19 may lead to new treatment strategies for reducing the morbidity of patients with this condition.

## Figures and Tables

**Table 1:** Clinical presentations and risk factors for severe human herpesviruses infections in immunocompromised/immunodeficient individuals (selected studies).

Human Herpesvirus	Clinical Presentation	Predisposing/Risk Factors
Herpes simplex viruses [13]	Herpes simplex virus encephalitis (type not differentiated)	HIV infection, malignancies, transplantation, immunosuppressive agents for connective tissue disorders
Herpes simplex virus 1 [14]	Stomatitis	Haematopoietic stem cell transplant for acute myeloid leukaemia
Varicella-zoster virus [15]	Herpes zoster/shingles	Autoimmune diseases, inflammatory bowel disease, chronic obstructive pulmonary disease, asthma, chronic kidney disease, depression, malignancies
Varicella-zoster Virus [16]	Meningitis/pneumonitis	Transplantation
Cytomegalovirus [17]	Retinitis	HIV infection
Cytomeglovirus [18]	Pneumonitis	Lung transplant
Epstein–Barr Virus [19]	Post-transplant lymphoproliferative disorder	Heart transplant
Epstein–Barr virus [20]	Haemophagocytic lymphohistiocytosis	Chronic active EBV infection
Human herpesvirus 6 (type not differentiated, but most likely 6B) [21]	Encephalitis	Leukaemia requiring haematopoietic stem cell transplant

**Table 3:** Selected case reports of herpes zoster following COVID-19 vaccination.

Case Report	Vaccine/Number of Cases	Description
Van Dam et al. [48]. Herpes zoster after COVID vaccination.	COVID-19 mRNA vaccine, 2 cases	Case 1. Female aged 29 years. Received 1st dose of the vaccine 8 January 2021 and observed grouped vesicles on 23 January. Rash not confirmed by VZV PCR. Case 2. Male aged 34 years. Received 1st dose of the vaccine 12 January 2021 and observed rash on right leg approximately two weeks later. Rash confirmed by VZV PCR.
Maruki et al. [49]. A case of varicella-zoster virus meningitis following BNT162b2 mRNA COVID-19 vaccination in an immunocompetent patient.	COVID-19 mRNA vaccine, 1 case	Female aged 71 years. Five days following her first COVID-19 vaccination, she developed a vesicular rash on the right side of her umbilicus and on her back. Later diagnosed with VZV meningitis.
Tanizaki and Miyamatsu [50]. Zoster sine herpete following BNT162b2 mRNA COVID-19 vaccination in an immunocompetent patient.	COVID-19 mRNA vaccine, 1 case	Male aged 60 years. Fever, fatigue, headache, cervical pain, and lumbar pain developed following a second dose of mRNA vaccine. Zoster sine herpete diagnosed on the basis of clinical presentation and VZV IgM positive serology.
Chiu et al. [51]. Herpes zoster following COVID-19 vaccine: a report of three cases.	COVID-19-modified adenovirus vaccine, 2 cases	Case 1. Male aged 46 years. Pain and itch over ipsilateral flank two days following receiving a first dose of vaccine. Typical rash developed later, but was not confirmed by PCR. Case 2. Male aged 42 years. Pain and itch over ipsilateral flank seven days following receiving a first dose of vaccine. Typical rash developed later, but was not confirmed by PCR.
Özdemir et al. [52]. Herpes zoster after inactivated SARS-CoV-2 vaccine in two healthy young adults.	SARS-CoV-2 inactivated vaccine, 2 cases	Case 1. Female aged 23 years. Reported itchy and painful rash on her back one day following vaccination with an inactivated SARS-CoV-2 vaccine. Rash not confirmed by PCR. Case 2. Male aged 21 years. Reported painful eruption of grouped vesicles on abdomen two days following vaccination with an inactivated SARS-CoV-2 vaccine. Rash not confirmed by PCR.
Daouk et al. [53]. Zoster meningitis in an immunocompetent child after COVID-19 vaccination, California, USA.	COVID-19 mRNA vaccine, 1 case	Report of live-attenuated varicella vaccine reactivation in an immunocompetent child after COVID-19 vaccination. Male aged 12 years. Onset of symptoms 11 days following COVID-19 vaccination. Severe flank and thigh pain for one week before the appearance of a papulovesicular rash and neurological symptoms. CSF and vesicular lesions tested positive for VZV by PCR.

PCR = polymerase chain reaction, CSF = cerebrospinal fluid.

## Data Availability

Not applicable.

## References

[1] Docherty A.B., Harrison E.M., Green C.A., Hardwick H.E., Pius R., Norman L., Holden K.A., Read J.M., Dondelinger F., Carson G. (2020). Features of 20133 UK patients in hospital with covid-19 using the ISARIC WHO clinical characterisation protocol: Prospective observational cohort study. BMJ.

[2] Del Rio C., Malani P.N. (2020). 2019 Novel Coronavirus-Important information for clinicians. JAMA.

[3] Huang C., Wang Y., Li X., Ren L., Zhao J., Hu Y., Zhang L., Fan G., Xu J., Gu X. (2020). Clinical features of patients infected with 2019 novel coronavirus in Wuhan, China. Lancet.

[4] Macera M., de Angelis G., Sagnelli C., Coppola N., Vanvitelli COVID-19 Group (2020). Clinical presentation of COVID-19, case series and review of the literature. Int. J. Environ. Res. Public Health.

[5] Schultz J.M., Perlin A., Saltzman R.G., Espinel Z., Galea S. (2020). Pandemic March: 2019 Coronavirus disease’s first wave circumnavigates the Globe. Disaster Med. Public Health Prep..

[6] Flores-Vega V.R., Monroy-Molina J.V., Jiménez-Hernández L.E., Torres A.G., Santos-Preciado J.I., Rosales-Reyes R. (2022). SARS-CoV-2, Evolution and emergence of new viral variants. Viruses.

[7] Prévost J., Finzi A. (2021). The great escape? SARS-CoV-2 variants evading neutralizing responses. Cell Host Microbe.

[8] Bian L., Gao F., Zhang J., He Q., Mao Q., Xu M., Liang Z. (2021). Effects of SARS-CoV-2 variants on vaccine efficacy and response strategies. Expert Rev. Vaccines.

[9] Fiolet T., Kherabi Y., MacDonald C.-J., Ghosn J., Peiffer-Smadja N. (2022). Comparing COVID-19 vaccines for their characteristics, efficacy and effectiveness against SARS-CoV-2 and variants of concern: A narrative review. Clin. Microbiol. Infect..

[10] Marks P., Woodcock J., Califf R. (2022). COVID-19 vaccination-becoming part of the new normal. JAMA.

[11] Walker P.J., Siddell S.G., Lefkowitz E.J., Mushegian A.R., Dempsey D.M., Dutilh B.E., Harrach B., Harrison R.L., Hendrickson R.C., Junglen S. (2019). Changes to virus taxonomy and the International Code of Virus Classification and Nomenclature ratified by the International Committee on taxonomy of Viruses (2019). Arch. Virol..

[12] Weidner-Glunde M., Kruminis-Kaszkiel E., Savanagouder M. (2020). Herpesviral latency-common themes. Pathogens.

[13] Tan I.L., McArthur J.C., Venkatesan A., Nath A. (2012). Atypical manifestations and poor outcome of herpes simplex encephalitis in the immunocompromised. Neurology.

[14] Heidenreich D., Krell S., Mueller N., Jawhar M., Nolte F., Hofmann W.-K., Klein S.A. (2020). Topical treatment of acyclovir-resistant herpes simplex virus stomatitis after allogeneic hematopoietic cell transplantation. Oncol. Res. Treat..

[15] Forbes H.J., Bhaskaran K., Thomas S.L., Smeeth L., Clayton T., Langan S.M. (2014). Quantification of risk factors for herpes zoster: Population based case-control study. BMJ.

[16] Takahashi Y., Hara S., Hoshiba R., Hibino S., Ito K., Zoshima T., Suzuki Y., Inoue D., Mizushima I., Fujii H. (2021). Pneumonia and central nervous system infection caused by reactivation of varicella-zoster virus in a living-donor kidney transplantation patient: Case report and review of the literature. CEN Case Rep..

[17] Tang Y., Sun J., He T., Shen Y., Liu L., Steinhart C.R., Chen J., Qi T., Wang Z., Song W. (2020). Clinical features of cytomegalovirus retinitis in HIV infected patients. Front. Cell. Infect. Microbiol..

[18] Santos C.A.G., Brennan D.C., Yusen R.D., Olsen M.A. (2015). Incidence, risk factors and outcomes of delayed-onset cytomegalovirus disease in a large retrospective cohort of lung transplant recipients. Transplantation.

[19] Asleh R., Alnsasra H., Habermann T., Briasoulis A., Kushwaha S.S. (2022). Post-transplant lymphoproliferative disorder following cardiac transplantation. Front. Cardiovasc. Med..

[20] He X., Wang J., Song D., Wang Z. (2022). Development of a nomogram to predict the risk of chronic active Epstein-Barr virus infection progressing to hemophagocytic lymphohistiocytosis. Front. Med..

[21] Berzero G., Campanini G., Vegezzi E., Paoletti M., Pichiecchio A., Simoncelli A.M., Colombo A.A., Bernasconi P., Borsani O., Di Matteo A. (2021). Human herpesvirus 6 encephalitis in immunocompetent and immunocompromised hosts. Neurol. Neuroimmunol. Neuroinflamm..

[22] Tay M.Z., Poh C.M., Rénia L., MacAry P.A., Ng L.F.P. (2020). The trinity of COVID-19, immunity, inflammation and intervention. Nat. Rev. Immunol..

[23] Iadecola C., Anrather J., Kamel H. (2020). Effects of COVID-19 on the nervous system. Cell.

[24] Meyerowitz E.A., Richterman A., Bogoch I.I., Low N., Cevik M. (2021). Towards an accurate and systematic characterisation of persistently asymptomatic infection with SARS-CoV-2. Lancet Infect. Dis..

[25] Goldman M. (2022). Long Covid, a great imitator of the 21th century. Front. Med..

[26] Giacobbe D.R., Di Bella S., Lovecchio A., Ball L., De Maria A., Vena A., Bruzzone B., Icardi G., Pelosi P., Luzzati R. (2022). Herpes simplex virus 1 (HSV-1) reactivation in critically ill COVID-19 patients: A brief narrative review. Infect. Dis. Ther..

[27] Luyt C.-E., Burrel S., Mokrani D., Pineton de Chambrun M., Luyt D., Chommeloux J., Guiraud V., Bréchot N., Schmidt M., Hekimian G. (2022). Herpesviridae lung reactivation and infection in patients with severe COVID-19 or influenza virus pneumonia: A comparative study. Ann. Intensive Care.

[28] Meyer A., Buetti N., Houhou-Fidouh N., Patrier J., Abdel-Nabey M., Jaquet P., Presente S., Girard T., Sayagh F., Ruckly S. (2021). HSV-1 reactivation is associated with an increased risk of mortality and pneumonia in critically ill COVID-19 patients. Crit. Care.

[29] Maia C.M.F., Marques N.P., De Lucena E.H.G., De Rezende L.F., Martelli D.R.B., Martelli-Júnior H. (2021). Increased number of herpes zoster cases in Brazil related to the COVID-19 pandemic. Int. J. Infect. Dis..

[30] Algaadi S.A. (2022). Herpes zoster and COVID-19 infection: A coincidence or a causal relationship?. Infection.

[31] Almutairi N., Almutairi A.N., Almazyad M., Alwazzan S. (2022). Herpes zoster in the era of COVID-19, a prospective observational study to probe the association of herpes zoster with COVID 19 infection and vaccination. Dermatol. Ther..

[32] Gatto I., Biagioni E., Coloretti I., Farinelli C., Avoni C., Caciagli V., Busani S., Sarti M., Pecorari M., Gennari W. (2022). Cytomegalovirus blood reactivation in COVID-19 critically ill patients: Risk factors and impact on mortality. Intensive Care Med..

[33] Taherifard E., Mortazavi R., Mokhtari M., Taherifard A., Salmi S.K., Taherifard E. (2022). Cytomegalovirus gastritis in a patient with severe acute respiratory syndrome coronavirus 2 infection: A case report and literature review. Respir. Med. Case Rep..

[34] Alanio C., Verma A., Mathew D., Gouma S., Liang G., Dunn T., Oldridge D.A., Weaver J., Kuri-Cervantes L., Pampena M.B. (2022). Cytomegalovirus latent infection is associated with an increased risk of COVID-19-related hospitalization. J. Infect. Dis..

[35] Weber S., Kehl V., Erber J., Wagner K.I., Jetzlsperger A.M., Burrell T., Schober K., Schommers P., Augustin M., Crowell C.S. (2022). CMV seropositivity is a potential novel risk factor for severe COVID-19 in non-geriatric patients. PLoS ONE.

[36] Pius-Sadowska E., Niedźwiedź A., Kulig P., Baumert B., Sobuś A., Rogińska D., Łuczkowska K., Ulańczyk Z., Wnęk S., Karolak I. (2022). CXCL8, CCL2, and CMV seropositivity as new prognostic factors for a severe COVID-19 course. Int. J. Mol. Sci..

[37] Meng M., Zhang S., Dong X., Sun W., Deng Y., Li W., Li R., Annane D., Wu Z., Chen D. (2022). COVID-19 associated EBV reactivation and effects of ganciclovir treatment. Immune. Inflamm. Dis..

[38] Shafiee A., Aghajanian S., Athar M.M.T., Gargari O.K. (2022). Epstein-Barr virus and COVID-19. J. Med. Virol..

[39] Chen T., Song J., Liu H., Zheng H., Chen C. (2021). Positive Epstein-Barr virus detection in coronavirus disease 2019 (COVID-19) patients. Sci. Rep..

[40] Xie Y., Cao S., Dong H., Lv H., Teng X., Zhang J., Wang T., Zhang X., Qin Y., Chai Y. (2021). Clinical characteristics and outcomes of critically ill patients with acute COVID-19 with Epstein-Barr virus reactivation. BMC Infect. Dis..

[41] Brooks B., Tancredi C., Song Y., Mogus A.T., Huang M.L.W., Zhu H., Phan T.L., Zhu H., Kadl A., Woodfolk J. (2022). Epstein-Barr virus and human herpesvirus-6 reactivation in acute COVID-19 patients. Viruses.

[42] Lino K., Alves L.S., Raposo J.V., Medeiros T., Souza C.F., da Silva A.A., de Paula V.S., Almeida J.R. (2022). Presence and clinical impact of human herpesvirus-6 infection in patients with moderate to critical coronavirus disease-19. J. Med. Virol..

[43] Nalbandian A., Sehgal K., Gupta A., Madhavan M.V., McGroder C., Stevens J.S., Cook J.R., Nordvig A.S., Shalev D., Sehrawat T.S. (2021). Post-acute COVID-19 syndrome. Nat. Med..

[44] Zubair A.S., McAlpine L.S., Gardin T., Farhadian S., Kuruvilla D.E., Spudich S. (2020). Neuropathogenesis and neurologic manifestations of the coronaviruses in the age of coronavirus disease 2019, a review. JAMA Neurol..

[45] Monje M., Iwasaki A. (2022). The neurobiology of long COVID. Neuron.

[46] Gold J.E., Okyay R.A., Licht W.E., Hurley D.J. (2021). Investigation of long COVID prevalence and its relationship to Epstein-Barr virus reactivation. Pathogens.

[47] Rohrhofer J., Graninger M., Lettenmaier L., Schweighardt J., Gentile S.A., Koidl L., Ret D., Stingl M., Puchhammer-Stöckl E., Untersmayr E. (2023). Association between Epstein-Barr virus reactivation and development of long-COVID fatigue. Allergy.

[48] Van Dam C.S., Lede I., Schaar J., Al-Dulaimy M., Rösken R., Smits M. (2021). Herpes zoster after COVID vaccination. Int. J. Infect. Dis..

[49] Maruki T., Ishikane M., Suzuki T., Uguii M., Katano H., Ohmagari N. (2021). A case of varicella zoster virus meningitis following BNT162b2 mRNA COVID-19 vaccination in an immunocompetent patient. Int. J. Infect. Dis..

[50] Tanizaki R., Miyamatsu Y. (2022). Zoster sine herpete following BNT162b2 mRNA COVID-19 vaccination in an immunocompetent patient. IDCases.

[51] Chiu H.H., Wei K.C., Chen A., Wang W.H. (2021). Herpes zoster following COVID-19 vaccine: A report of three cases. QJM.

[52] Özdemir A.K., Kayhan S., Çakmak S.K. (2021). Herpes zoster after inactivated SARS-CoV-2 vaccine in two healthy young adults. J. Eur. Acad. Dermatol. Venereol..

[53] Daouk S.K., Kamau E., Adachi K., Aldrovandi G.M. (2022). Zoster meningitis in an immunocompetent child after COVID-19 vaccination, California, USA. Emerg. Infect. Dis..

[54] Hertel M., Heiland M., Nahles S., Von Laffert M., Mura C., Bourne P.E., Preissner R., Preissner S. (2022). Real-world evidence from over one million COVID-19 vaccinations is consistent with reactivation of the varicella-zoster virus. J. Eur. Acad. Dermatol. Venereol..

[55] Fathy R.A., McMahon D.E., Lee C., Chamberlin G.C., Rosenbach M., Lipoff J.B., Tyagi A., Desai S.R., French L.E., Lim H.W. (2022). Varicella-zoster and herpes simplex virus reactivation post-COVID-19 vaccination: A review of 40 cases in an International Dermatology Registry. J. Eur. Acad. Dermatol. Venereol..

[56] Gringeri M., Battini V., Cammarata G., Mosini G., Guarnieri G., Leoni C., Pozzi M., Radice S., Clementi E., Carnovale C. (2022). Herpes zoster and simplex reactivation following COVID-19 vaccination: New insights from a vaccine adverse event reporting system (VAERS) database analysis. Expert Rev. Vaccines.

[57] Abu-Rumeileh S., Mayer B., Still V., Tumani H., Otto M., Senel M. (2022). Varicella zoster virus-induced neurological disease after COVID-19 vaccination: A retrospective monocentric study. J. Neurol..

[58] Martinez-Reviejo R., Tejada S., Adebanjo G.A.R., Chello C., Machado M.C., Parisella F.R., Campins M., Tammaro A., Rello J. (2022). Varicella-Zoster virus reactivation following severe acute respiratory syndrome coronavirus 2 vaccination or infection: New insights. Eur. J. Intern. Med..

[59] Akpandak I., Miller D.C., Sun Y., Arnold B.F., Kelly J.D., Acharya N.R. (2022). Assessment of herpes zoster risk among recipients of COVID-19 vaccine. JAMA Netw. Open.

[60] Busnadiego I., Abela I.A., Frey P.M., Hofmaenner D.A., Scheier T.C., Schuepbach R.A., Buehier P.K., Brugger S.D., Hale B.G. (2022). Critically ill COVID-19 patients with neutralizing autoantibodies against type 1 interferons have increased risk of herpesvirus disease. PLoS Biol..

[61] Peluso M.J., Deveau T.M., Munter S.E., Ryder D.M., Buck A.M., Beck-Engeser G., Chan F., Lu S., Goldberg S.A., Hoh R. (2022). Impact of pre-existing chronic viral infection and reactivation on the development of long COVID. J. Clin. Investig..

[62] Davitt E., Davitt C., Mazer M.B., Areti S.S., Hotchkiss R.S., Remy K.E. (2022). COVID-19 disease and immune dysregulation. Best Pract. Res. Clin. Haematol..

[63] Bjornevik K., Cortese M., Healy B.C., Kuhle J., Mina M.J., Leng Y., Elledge S.J., Niebuhr D.W., Scher A.I., Munger K.L. (2022). Longitudinal analysis reveals high prevalence of Epstein-Barr virus associated with multiple sclerosis. Science.

[64] Dotan A., Muller S., Kanduc D., David P., Halpert G., Shoenfeld Y. (2021). The SARS-CoV-2 as an instrumental trigger of autoimmunity. Autoimmun. Rev..

[65] Bellucci G., Rinaldi V., Buscarino M.C., Reniè R., Bigi R., Pellicciari G., Morena E., Romano C., Marrone A., Mechelli R. (2021). Multiple sclerosis and SARS-CoV-2, Has the interplay started?. Front. Immunol..

[66] Wang Q., Iketani S., Li Z., Liu L., Guo Y., Huang Y., Bowen A.D., Liu M., Wang M., Yu J. (2023). Alarming antibody evasion properties of rising SARS-CoV-2 BQ and XBB subvariants. Cell.

[67] Stein S.R., Ramelli S.C., Grazioli S., Chung J.Y., Singh M., Yinda C.K., Winkler C.W., Sun J., Dickey J.M., Ylaya K. (2022). SARS-CoV-2 infection and persistence in the human body and brain at autopsy. Nature.

[68] Wang L., Davis P.B., Volkow N.D., Berger N.A., Kaelber D.C., Xu R. (2022). Association of COVID-19 with new-onset Alzheimer’s disease. J. Alzheimer’s Dis..

[69] Itzhaki R.F. (2022). COVID-19 and Alzheimer’s Dis: What is the connection?. J. Alzheimer’s Dis..

[70] Marks P.W., Gruppuso P.A., Adashi E.Y. (2023). Urgent need for next-generation COVID-19 vaccines. JAMA.

